# Evaluation of secretomes derived from human dermal and adipose tissue mesenchymal stem/stromal cells for skin wound healing: not as effective as cells

**DOI:** 10.1186/s13287-023-03630-y

**Published:** 2024-01-17

**Authors:** Helena Debiazi Zomer, Victor Juan de Souza Lima, Monique Coelho Bion, Karynne Nazare Lins Brito, Michele Rode, Marco Augusto Stimamiglio, Talita da Silva Jeremias, Andrea Gonçalves Trentin

**Affiliations:** 1 Department of Physiological Sciences, University of Florida, Gainesville, USA; 2 Department of Cell Biology, Embryology, and Genetics, Federal University of Santa Catarina, Florianópolis, Brazil; 3 National Institute of Translational Neuroscience, Federal University of Rio de Janeiro, Rio de Janeiro, Brazil; 4 Laboratory for Stem Cells Basic Biology, Carlos Chagas Institute, FIOCRUZ/PR, Curitiba, Paraná Brazil; 5 National Institute of Science and Technology for Regenerative Medicine, Rio de Janeiro, Brazil

**Keywords:** MSC, Dermis, Conditioned medium, Exosomes, Integra matrix, Proteomic

## Abstract

**Background:**

Although the paracrine effects of mesenchymal stem/stromal cells (MSCs) have been recognized as crucial mediators of their regenerative effects on tissue repair, the potential of MSC secretomes as effective substitutes for cellular therapies remains underexplored.

**Methods:**

In this study, we compared MSCs from the human dermis (DSCs) and adipose tissue (ASCs) with their secretomes regarding their efficacy for skin wound healing using a translationally relevant murine model.

**Results:**

Proteomic analysis revealed that while there was a substantial overlap in protein composition between DSC and ASC secretomes, specific proteins associated with wound healing and angiogenesis were differentially expressed. Despite a similar angiogenic potential in vivo, DSC and ASC secretomes were found to be less effective than cells in accelerating wound closure and promoting tissue remodeling.

**Conclusions:**

Overall, secretome-treated groups showed intermediary results between cells- and control-treated (empty scaffold) groups. These findings highlight that although secretomes possess therapeutic potential, their efficacy might be limited compared to cellular therapies. This study contributes to the growing understanding of MSC secretomes, emphasizes the need for further protocol optimization, and offers insights into their potential applications in regenerative medicine.

**Supplementary Information:**

The online version contains supplementary material available at 10.1186/s13287-023-03630-y.

## Introduction

Mesenchymal stem/stromal cells (MSCs) are multipotent stem cells present in virtually all adult tissues that participate in homeostasis and repair by the secretion of paracrine factors and differentiation in tissue-specific cells [1–4]. MSCs can be isolated with high yield and maintain their proliferative capacity and genetic stability for several passages in vitro [5–7]. They are classified as low or non-immunogenic as they express low levels of MHC I and lack MHC II and other co-stimulatory molecules. They do not cause T-cell activation and consequent rejection when allogeneically or xenogeneically transplanted [8–10], and due to their limited plasticity, they do not form tumors in vivo [9–12]. MSCs have been tested for multiple therapeutic applications in regenerative medicine, including neurodegenerative disorders, renal failure, diabetes, and skin wound healing [4, 13–16]. Their ability to promote cell growth, angiogenesis, and immunomodulation has been widely described and supported by basic, pre-clinical, and clinical studies [16–19].

We have previously isolated and characterized MSCs derived from the human dermis and adipose tissue (DSCs and ASCs, respectively) harvested from tissues discarded after elective abdominoplasties [5, 7, 20]. We have shown that both DSCs and ASCs share the general MSC immunophenotype (CD73+, CD90+, CD105+, CD34−, and CD45−) and mesodermal differentiation potential (adipogenic, osteogenic, and chondrogenic). Both have similar self-renewal capacity and remain genetically stable over time in culture with a low frequency of nuclear alterations. However, while the isolation procedure for ASCs recovers a higher initial number of cells, the DSCs show shorter doubling time/faster proliferation and quicker closure of in vitro wounds as evaluated by cell scratch assay [7]. We also tested DSCs and ASCs’ potential to promote skin wound healing in a mouse model, using a commercially available collagen-based dermal template (Integra bilayer wound dressing, Integra LifeSciences) as a delivery system. We showed that animals treated with either DSC or ASC have accelerated wound closure in comparison to controls (empty scaffold) [21–23]. On day 3 (inflammatory phase of wound healing), both DSC and ASC modulated the polarization of macrophages to a pro-repair phenotype, and on day 7 (proliferative phase), they promoted graft integration and increased angiogenesis in comparison to controls [13]. As graft integration is a crucial factor when predicting the success of healing, our findings indicate that the association of MSCs to FDA-approved dermal templates could avoid recurring graft complications leading to detachment. Additionally, at a late stage of remodeling (day 60), MSC-treated animals showed neoepidermis and collagen deposition similar to normal skin and the presence of regenerated hair follicles and elastic fibers. Notably, animals treated with DSCs had a denser collagen network and smaller visible scars than ASC and control-treated animals [13]. Overall, our work highlighted the dermis as a promising source of MSCs for regenerative therapies for skin wound healing, a finding recently validated in clinical settings by others [21–23].

Paradoxically, it has been extensively demonstrated that MSCs do not persist long-term in the host organism after transplantation [17, 24–26]. It is now widely accepted that MSCs act mainly through paracrine effects rather than direct differentiation into tissue-specific cells [27–29]. This paradigm shift opened a new, unexplored realm of cell-free possibilities for regenerative medicine. Acellular compounds have facilitated clinical handling, can be produced on a large scale to be readily available, and can be evaluated as conventional pharmaceutical agents [30–32]. The therapeutic application of MSCs’ paracrine factors—their secretome—is safer than cell transplantation as it removes the risk for tumorigenesis and significantly reduces immunogenicity [28, 32–34]. Secretomes consist of various bioactive molecules, including soluble proteins, lipids, extracellular vesicles, and nucleic acids [35–37]. In the last decade, studies have focused on how to use the MSCs secretome (or their isolated components) therapeutically. We have already learned that secretomes share, to some extent, the immunoregulatory and angiogenic properties of their originating cells in various diseases and conditions [38–40]. Our own work showed that the secretome of DSC and ASC (seDSC and seASC, respectively) promotes in vitro wound closure of human dermal fibroblasts and keratinocytes and vascular network formation on human umbilical vein endothelial cells (HUVECs) [7]. As observed in our cell study, dermal-derived secretomes showed significantly better results than those derived from the adipose tissue, reinforcing the dermis as the ideal source of cells and bioactive molecules for skin wound healing applications.

Despite current research pointing toward an advantage of secretome therapies over cellular therapies, the vast majority of studies have compared secretomes or isolated components against untreated controls rather than the cells they derive from [36, 41–43]. Thus, this study aimed to determine whether MSC secretomes can be effectively used in substitution for cellular therapies. Herein, we evaluated the protein composition of dermal and adipose-derived MSC secretomes and their potential to induce skin wound healing in a mouse model compared to cells and empty scaffold controls.

## Methods

### Isolation and culture of DSCs and ASCs

DSCs and ASCs were obtained as we previously described [13]. They were extracted from sections of human skin and subcutaneous adipose tissue after written informed consent obtained from healthy patients (*n* = 6) undergoing abdominoplasty, aged between 22 and 55 years. The research protocol was approved by the Ethics Committee of the Federal University of Santa Catarina, Brazil (1.076.626). Briefly, to extract MSCs from the subcutaneous adipose tissue or dermis, sections were incubated with collagenase type I (1%, 1 h, 37 °C; Sigma-Aldrich) or trypsin–EDTA (0.25%, 1 h, 37 °C; Sigma-Aldrich), respectively. Enzymatic digestions were stopped by adding 10% fetal bovine serum (FBS, Vitrocell). The resulting cell suspensions were filtered through a 70-μm mesh (BD Bioscience) and centrifuged (300 g, 5 min, 22 °C). Cell pellets were then resuspended in an erythrocyte lysis solution (155 mM NH4Cl, 12 mM NaHCO3, and 0.1 mM EDTA) and centrifuged again. After discarding the supernatants, the pellets were resuspended in a complete medium of Dulbecco's Modified Eagle's Medium (DMEM) supplemented with 10% FBS and 1% Penicillin–Streptomycin (Gibco). Cells were seeded in 25 cm^2^ culture flasks (Corning) and maintained at 37 °C in a humidified atmosphere with 5% CO_2_. The medium was changed every three days until the cells reached 90% confluence. The DSCs and ASCs obtained were previously characterized by our research group, as described in Zomer et al. [7].

### Preparation of seDSCs and seASCs

The secretomes were obtained following a protocol adapted from Fong et al. [44] and previously described by our group [7]. In summary, DSCs or ASCs monolayers at 90% confluence were subjected to a triple wash with PBS and maintained in DMEM without FBS for 48 h. Cell supernatants were collected and processed by centrifugation (5 min, 300 g) to pellet and exclude eventual floating dead cells, and then filtered through a 0.22 µm filter to remove debris. The resulting secretome was concentrated tenfold using an Amicon Ultra-15-Centrifugal Filter 3 kDa MWCO (Millipore) by centrifugation (45 min, 5,000 g), following the manufacturer's instructions. The concentrated secretome was aliquoted and stored at − 80 °C for up to 4 months for downstream applications. The total protein content of the secretome was quantified using the DC Protein kit (Bio-Rad) according to the manufacturer’s protocol, and reading was carried out using the Tecan Infinite M200 microplate reader. Total protein secreted was similar in seDSC and seASC (average of 194.4 and 209.4 µg per 10^6^ cells, respectively). Secretomes were diluted to a final concentration of 1 µg/µl and wound healing studies used a dosage of 200 µg of total protein per wound, in reference to cell group controls that received 10^6^ DSC or ASC per wound.

### Proteomic analysis

The proteomic analysis of seDSC and seASC was conducted using data generated by the Mass Spectrometry Platform (RPT02H) located at Instituto Carlos Chagas—Fiocruz (Brazil). The liquid chromatography with tandem mass spectrometry (LC–MS/MS) method was employed for this study. In summary, 5 µg of peptides from each sample was analyzed in triplicate using the Thermo Scientific Easy-nLC 1000 liquid chromatography system coupled to an LTQ Orbitrap XL ETD mass spectrometer. Protein identification, quantification, and validation were conducted using the MaxQuant Platform. Data analysis was performed using the Perseus software. Proteins identified with a minimum of two unique peptides in at least three samples were selected for analysis. Gene Ontology (GO) analysis was conducted using the g: Profiler bioinformatic tool (https://biit.cs.ut.ee/gprofiler/gost) [45], and the most relevant terms were presented (*p* < 0.001).

### Animals

The Animal Ethics Committee of the Federal University of Santa Catarina, Brazil (P. 00810), approved all animal procedures. Female and male C57BL/6 mice 4 to 6 months old and weighing between 20 to 30 g were utilized in the study. The mice had unrestricted access to standard chow and drinking water and were housed under a 12-h light/dark cycle. This study adheres to the ARRIVE guideline for the reporting of animal experiments. The wound healing procedure and evaluation are outlined below.

### Surgical procedure

Surgical procedures were performed under aseptic conditions in the animal facility operating room, following our previously described protocol [13]. Mice were anesthetized with intraperitoneal injections of ketamine (100 mg/kg, Vetnil) and xylazine (10 mg/kg, Syntec). Before the surgery, the dorsum of the animals was shaved, and a critical-sized, full-thickness round skin wound measuring 1.4 cm in diameter was excised. The mice were randomly divided into five groups, each containing 6–8 animals (total animals used: 70), as defined by power analysis based on our previous study [13]. Equal numbers of males and females were used per group. Integra matrix was used as a delivery system. Integra is a bilayer biomaterial composed of a temporary silicone layer (“epidermal” component) and a degradable bovine collagen and shark chondroitin-6-sulfate matrix (“dermal” component). While the dermal component provides a tridimensional structure that act as a scaffold and a sponge to hold cells and secretomes, the material’s silicone layer act as a stent, effectively stabilizing the wounds to prevent premature contraction observed in murine wounds, therefore facilitating the formation of significant granulation tissue and better mirroring human healing [47]. Empty fragments of Integra matrix (negative control), pre-seeded for 24 h with 1 × 10^6^ DSCs or ASCs (cell-treated groups), or soaked in 100 µl of seDSC or seASC (secretome-treated groups) were sutured to the surrounding skin using eight equidistant stitches of 6.0 nylon suture. A combination of cells or secretomes from three donors was used per mouse to minimize individual variations. Additionally, for secretome-treated groups, 100 µl of secretomes was injected intradermically around the wound edges. No additional dressing was applied after the procedure, as the biomaterial silicone layer protected against dehydration and contamination. Post-operative analgesia was administered for 3 days using acetaminophen (1 mg/ml) in the drinking water. There were no signs of infection, so antibiotics were not used. The animals were individually housed until euthanasia, which was performed by isoflurane overdose on days 7 and 21 after the surgery.

### Clinical evaluation of wound healing

The animals were monitored daily to assess graft appearance, wound closure, and time of Integra silicone layer detachment. Immediately following the surgery and euthanasia, the mice were weighed, and the wounds or scars were measured using a caliper ruler and documented through photography. Any progressive weight loss or observable behavioral changes (e.g., decreased motor activity, altered eating patterns, vocalization, self-mutilation, or piloerection) were considered humane endpoints for euthanasia and would result in the exclusion of the animals from the study. No animals were excluded from the study.

### Histopathological analysis

After euthanasia, the wound area was carefully excised, halved, and then fixed in a 4% paraformaldehyde solution for 24 h. Subsequently, all tissue samples underwent standard histological processing techniques. The tissues were embedded in paraffin wax, and 5 µm sections were prepared and mounted on glass slides. The sections were deparaffinized and subjected to staining with hematoxylin and eosin.

### Proliferative response and vascular density

In order to assess the proliferative phase of healing, the thickness of the granulation tissue and the number of blood vessels were measured on histological samples collected on day 7 after surgery under a magnification of 400x, as previously described [13]. Six random fields of the granulation tissue were measured in the wound area from the subcutaneous adipose tissue to the inferior border of the graft, and four fields in the adjacent skin area were recorded as a control of intact dermal thickness. The density of blood vessels was counted in six random fields within the granulation tissue and in eight fields of normal skin for reference. Images were captured using an optical microscope (Olympus BX41) and a digital sight camera (Olympus SC30).

### Reepithelization and matrix remodeling

Neoepidermis thickness and graft remodeling (Integra collagen layer) were evaluated on histological sections at a magnification of 400 × in tissues collected on day 21 after surgery, as previously described [13]. To measure neoepidermis thickness, six random fields in the scar/wound area and six in the adjacent intact skin (intact epidermal thickness control) were selected. The measurements were taken perpendicularly to the skin surface. Graft remodeling of the Integra collagen layer was evaluated based on the percentage of the visible non-degraded matrix within the scar/wound area. The scoring system used was as follows: score 1 represented 0% to 25% remodeling, score 2 indicated 26% to 50% remodeling, score 3 denoted 51% to 75% remodeling, and score 4 indicated 76% to 100% remodeling [13].

#### Statistical analysis

The statistical significance was assessed using Student’s *t* test (control vs. treated groups; cells vs. secretomes groups). GraphPad Prism software was utilized for these analyses when appropriate. Each experiment had a minimum of 6 biological replicates (6 mice). Data are presented as mean ± standard deviation (SD), and differences were considered significant when *p* < 0.05.

## Results

### DSC and ASC differentially secrete proteins with roles in skin wound healing

**Fig. 1:**
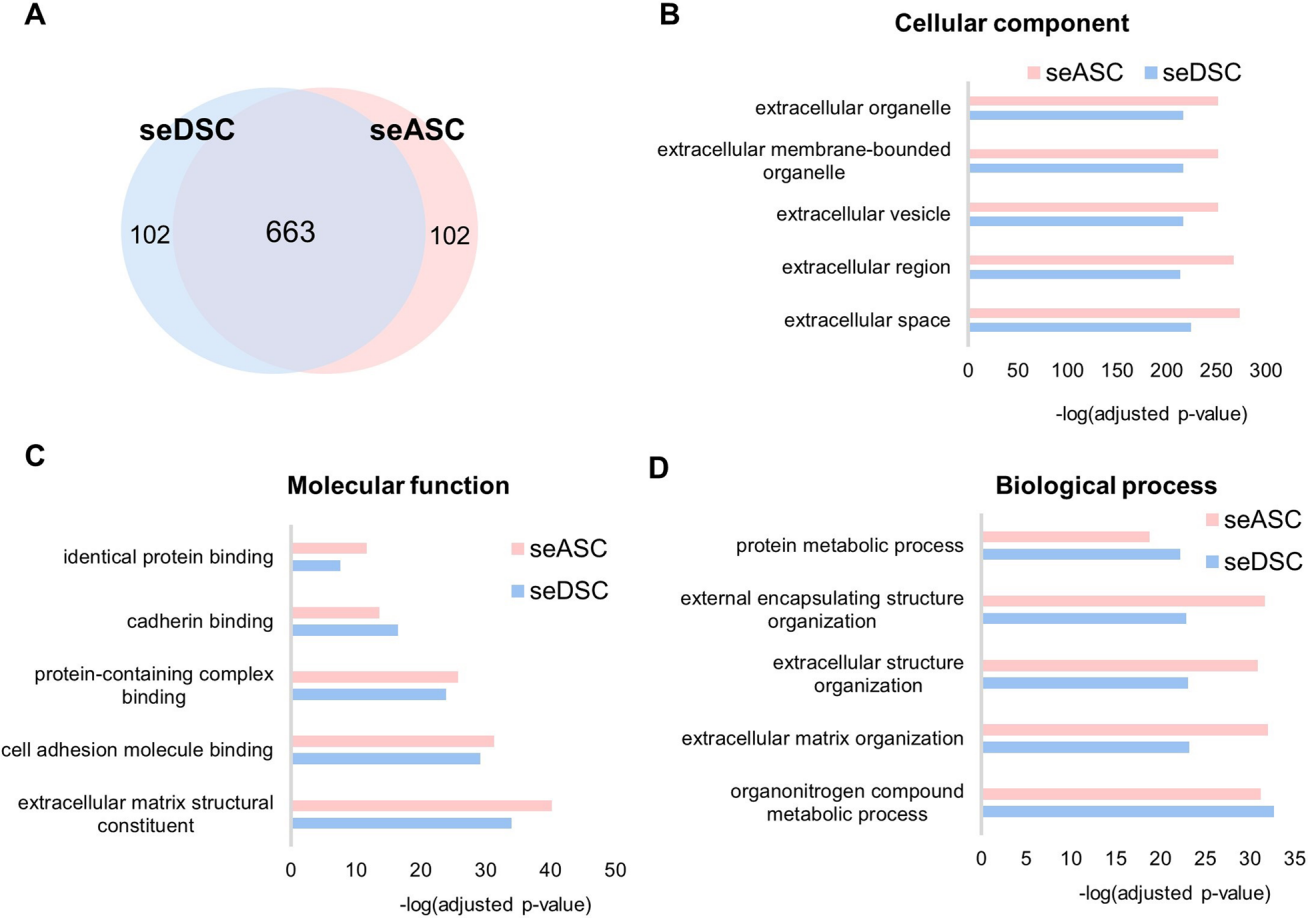
Comparative proteomic analysis of ASC and DSC secretomes. **A** Venn diagram representing the number of proteins identified in both secretome and the exclusively expressed in each group. In **B**–**D**, bar graphs illustrate the top five enriched GO terms in seDSC and seASC for cellular components (**B**), molecular functions (**C**), and biological processes (**D**)

Proteomics analysis revealed that among the 867 identified proteins, 663 were found in both DSC and ASC secretomes (76.47%), indicating a significant overlap (Fig. 1A). Moreover, 102 proteins were exclusive of seDSC and another 102 exclusives of seASC. Protein distribution over cellular components was remarkably similar in both secretomes (Fig. 1B). Specifically, extracellular space proteins were enriched in both and accounted for 63.8% (488 proteins) in seDSC and 69.4% (531 proteins) in seASC. Gene ontology analysis of molecular functions (Fig. 1C) showed that both secretomes were enriched in proteins related to the extracellular matrix structural constituent, 8.7% (67 proteins) in seASC compared to 9.4% (72 proteins) in seDSC. The secretomes were also enriched in proteins involved in the same biological processes (Fig. 1D), especially in extracellular matrix organization, with 84 proteins in seASC and 75 in seDSC.

Several GO terms related to wound healing and vasculature were identified as enriched in both secretomes. Notably, seASC displayed a higher variety of proteins associated with vasculature development (94 proteins compared to 82 proteins in seDSC), blood vessel development (89 proteins compared to 78 proteins in seDSC), and angiogenesis (67 proteins compared to 58 proteins in seDSC). The complete list of proteins and GO terms found in seDSC and seASC are detailed in Additional file 1: Table S1. Differentially expressed proteins (*p* < 0.05) with a role in wound healing are described in Table 1.

**Table 1:** Differentially expressed proteins (*p* < 0.05) in seDSC and seASC with described roles on skin wound healing

Protein	seDSC	seASC	Function
A2M	–	++	Anti-protease. Inhibits fibrinolysis and coagulation processes
ABI3BP	++	+++	Inhibits proliferation and induces MSC differentiation
ANGPT1	++	–	Development, maturation, vascular stability, and angiogenesis
CCBE1	++	–	Angiogenesis and lymphoangiogenesis
CFL1	↑++	++	Cell polarity and migration
CHI3L1	+++	↑+++	Inflammation and tissue remodeling
COL15A1	++	↑++	Structural protein that stabilizes microvessels
COL5A1	+++	↑+++	Regulates the diameter of collagen fibrils
COMP	+++	↑+++	Increased in fibrotic scars; potential role in vascular remodeling
DDX42	++	–	Involved in cell viability
ECM2	–	++	Facilitates matrix organization and cellular adhesion
EIF3D	++	–	Regulates proliferation, differentiation, and apoptosis
FERMT2	++	↑++	Mediates focal adhesion. Participates in the connection between extracellular matrix and cytoskeleton, and modulates cellular morphology
GREM1/2	↑+	+	BMP antagonist. Stimulates viability, proliferation, and osteogenic and chondrogenic differentiation
HSPG2	++	+++	Regulates vascular response after injury. Anti-angiogenic action
IGFBP2	++	–	Inhibits IGF-dependent cell proliferation
IGFBP5	–	++	Controls cell survival, differentiation, and apoptosis
IL-6	++	+++	Can act as a pro- or anti-inflammatory cytokine. Immunomodulatory, angiogenic, and anti-apoptotic effects
IQGAP2	++	–	Interacts with the cytoskeleton, adhesion molecules, and signaling pathways to regulate cellular morphology and motility
LAMA1	–	++	Mediates cell adhesion, migration, and tissue organization
MMP9	++	+++	Degrades collagen IV and V, along with other extracellular proteins
NOV	++	↑++	Modulates proliferation, adhesion, migration, differentiation, and cell survival. Induces angiogenesis and acts as a receptor ligand for integrins. Stimulates fibroblast adhesion and chemotaxis and decreases the adherence of inflammatory monocytes. Suppresses MMP9 expression
PARP1	++	–	Modulates the expression of inflammatory genes and is involved in DNA repair, genomic stability, and apoptosis
PCDH7	++	–	Induces platelet degranulation, cell–cell recognition, and adhesion
PLAU	++	↑++	Functions as a protease in tissue remodeling and cell migration
PTGIS	–	++	Synthesizes prostaglandin I2, induces vasodilation, and inhibits platelet aggregation
RARRES2	–	++	Modulates inflammation, acts as a chemotactic agent for leukocytes, and exhibits antimicrobial effects on the skin. Induces adipogenesis and angiogenesis and regulates lipid and glucose metabolism
SBSN	–	++	Controls epidermal differentiation
SEMA7A	++	+++	Regulates cell migration and immune responses. Stimulates focal adhesion and the production of inflammatory cytokines
SERPINE1	+++	↑+++	Protease inhibitor
SERPINE2	↑+++	+++	Protease inhibitor
SLIT2	++	↑++	Inhibits migration, proliferation, chemotaxis, and angiogenesis
SPARC	+++	↑+++	Cell–matrix interactions. Stimulates MMP, angiogenesis, proliferation, and migration
TGFB1	+	++	Controls cell proliferation and differentiation
THBS2	+++	↑+++	Modulates adhesion and migration of mesenchymal cells
TIMP1	+++	↑+++	Inhibits MMP and apoptosis and promotes proliferation
VCAN	++	+++	Regulates cell motility, proliferation, and differentiation. May participate in intercellular signaling
VCL	↑+++	+++	Enhances cell adhesion

Scores are based on peak intensity label-free quantification

For proteins with similar scores but statistically different, arrows (↑) indicate in which secretome the expression was higher

The functions of the proteins were obtained through searches on the online platforms http://www.uniprot.org and http://www.ncbi.nlm.nih.gov/gene

LFQ: Label-free quantification; A2M: Alpha-2-macroglobulin; ABI3BP: ABI family member 3-binding protein; ANGPT1: Angiopoietin-1; BMP: Bone morphogenetic protein; CCBE1: Collagen and calcium-binding EGF domain-containing protein 1; CFL1: Cofilin-1; CHI3L1: Chitinase-3-like protein 1; COL15A1: Collagen alpha-1 (XV) chain – Restin 1,2,3,4; COL5A1: Collagen alpha-1 (V) chain; COMP: Cartilage oligomeric matrix protein; DDX42: ATP-dependent RNA helicase; ECM2: Extracellular matrix protein 2; EIF3D: Eukaryotic translation initiation factor 3 subunit D; FERMT2: Fermitin family homolog 2; GREM1/2: Gremlin 1/2; HSPG2: Heparin sulfate proteoglycan core protein; IGFBP: Insulin-like growth factor-binding protein; IL: Interleukin; IQGAP2: Ras GTPase-activating-like protein; LAMA1: Laminin alpha 1 subunit; MMP: Matrix metalloproteinase; NOV: Nephroblastoma overexpressed protein, also known as IGFBP-9; PARP1: Poly [ADP-ribose] polymerase 1; PCDH7: Protocadherin-7; PLAU: Urokinase-type plasminogen activator; PTGIS: Prostacyclin synthase; RARRES2: Retinoic acid receptor responder 2 protein; SBSN: Suprabasin; SEMA7A: Semaphorin-7A; SERPINE1: Serpin family E member 1, also known as PAI-1; SERPINE2: Serpin family E member 2; SLIT2: Slit homolog 2 protein; SPARC: Secreted protein acidic and rich in cysteine, also known as Osteonectin; TGFB1: Transforming growth factor beta 1; THBS2: Thrombospondin 2; TIMP1: Tissue inhibitor of metalloproteinases 1; VCAN: Versican core protein; VCL: Vinculin

### Secretomes are not as effective as cells in promoting wound closure

**Fig. 2:**
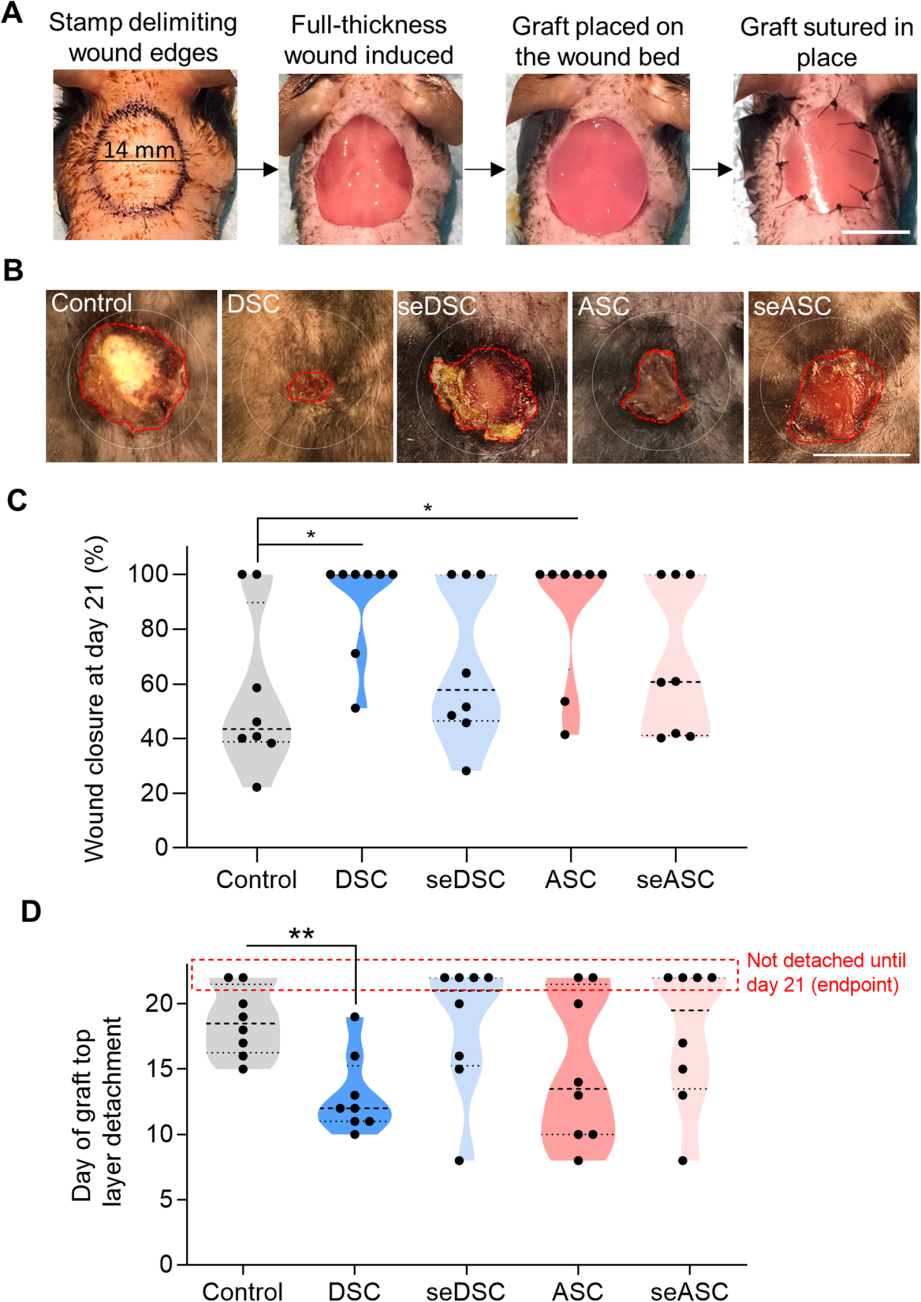
Secretomes are not as effective as cells in inducing wound closure. **A** Illustrative images depict the procedure of inducing full-thickness skin wounds in mice. **B** Representative images of wounds 21 days after the surgical procedure. The white dotted circle represents the initial size of the wounds, and the yellow lines highlight the final borders. Scale bar: 1 cm. **C** Violin plots presenting the percentage of animals with closed wounds on day 21. **D** Day of Integra silicone layer detachment. Silicone layers that did not detach by the study's endpoint (day of euthanasia) were considered day 22 for statistical purposes and are indicated by a red dotted rectangle. Each dot represents one animal evaluated. *p < 0.05 and **p < 0.01 by Student’s *t* test (control vs. each treated group; each cell vs. the respective secretome group)

Subsequently, the therapeutic potential of seDSC and seASC was evaluated in a mouse model of skin wound healing, using a collagen-based scaffold (Integra Matrix) as a delivery system, in comparison to the cells they derived from and the empty scaffold (negative control) (Fig. 2A). On day 21 after wounding, both DSC and ASC treatments resulted in accelerated wound closure in mice compared with the empty scaffold (DSC: 90.3% ± 18.7; ASC: 86.9% ± 24.5 versus control: 55.8% ± 29.1, both *p* < 0.05) (Fig. 2B, C). Secretome treatments similarly reached intermediary results in comparison to cells and empty scaffold control-treated groups (seDSC: 67.3% ± 28.8; seDSC 68.1% ± 27.7), with no statistically significant differences.

The Integra scaffold top silicone layer detachment was evaluated, as we have previously demonstrated a direct correlation between the timing of detachment and healing progress [13]. By day 21 (endpoint of this study), only the animals of the DSC-treated group had all silicone layers detached (Fig. 2D). In contrast, the silicone did not detach in two out of eight animals (25%) in the control and ASC-treated groups; and in 50% of both secretome-treated animals. On average, detachment occurred significantly earlier in the DSC-treated group than in control (DSC: day 13 ± 3 versus control: day 19 ± 3, *p* < 0.01), while neither of the other groups showed significant differences with each other. Together, these findings suggest that cell treatments are superior in accelerating wound closure compared to their respective secretomes.

### Similar angiogenic potential but thinner granulation tissue in secretome-treated wounds compared to cells

**Fig. 3:**
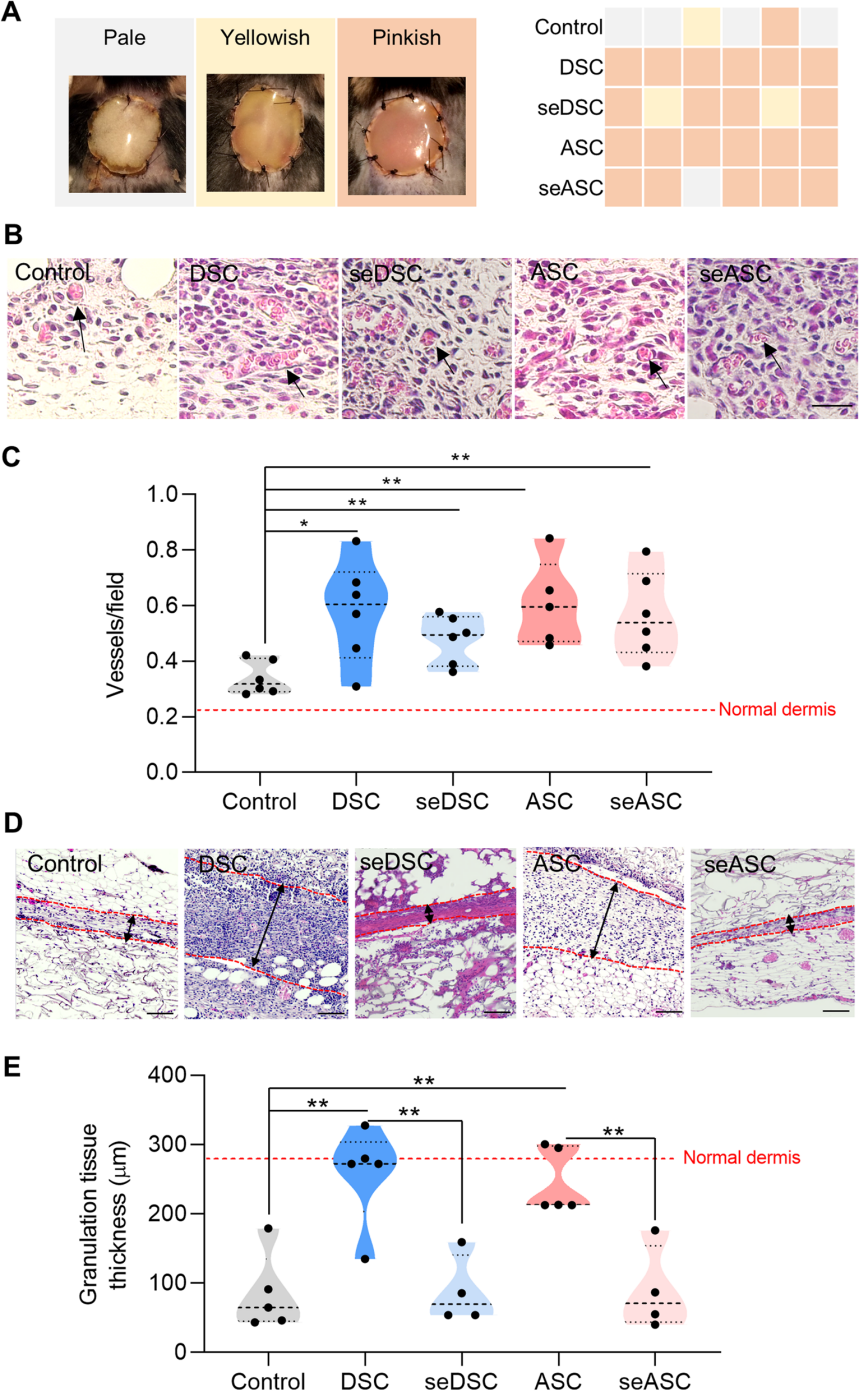
Assessment of the proliferative phase of healing in cell- and secretome-treated wounds. **A** Clinical aspect of the wound with Integra Matrix on day 7 post-wounding. The pinkish color indicates improved graft integration and increased angiogenesis, contrasting with the pale/yellowish appearance. **B** Representative images of blood vessels (indicated by arrows) within the granulation tissue. Scale bar: 50 µm. **C** Quantification of the number of blood vessels per field. Each data point represents the evaluation of one animal. The red dotted line indicates the blood vessel density in normal dermis. **D** Representative images of granulation tissue thickness. Scale bar: 100 µm. The red dotted line delineates the tissue borders, and the black arrows indicate its thickness. **E** Quantification of granulation tissue thickness. The red dotted line represents the thickness of the normal dermis. *p < 0.05 and **p < 0.01 by Student’s *t* test (control vs. treated groups; cells vs. secretomes groups)

The proliferative phase of wound healing is marked by the development of granulation tissue enriched in blood vessels and fibroblasts in the wound matrix [46, 47]. Clinically, a well-developed granulation tissue appears pinkish through the graft silicone layer from the high vascular density. In contrast, pale and yellowish colors correlate with poor graft integration (Fig. 3A) [13]. At day 7 post-wounding, all cell-treated wounds were pinkish, while in the empty scaffold controls, this was seen in only one out of six animals (16.6%). In seDSC and seASC-treated wounds, four of six (66%) and five of six (83%) grafts showed the expected pinkish color, respectively. Histological quantification of blood vessels confirmed a comparable, significant increase in vascular density in animals treated with secretomes and cells, in comparison to empty scaffold controls (Fig. 3B, C). However, cell-treated animals showed thicker granulation tissues (similar to the adjacent intact dermis) than secretome- and empty scaffold-treated groups (Fig. 3 D, E). These findings suggest that secretomes are as effective as cells in inducing angiogenesis but may not adequately promote fibroblast proliferation and collagen secretion in the proliferative healing phase.

### Delayed remodeling in secretome-treated wounds compared to cells

**Fig. 4:**
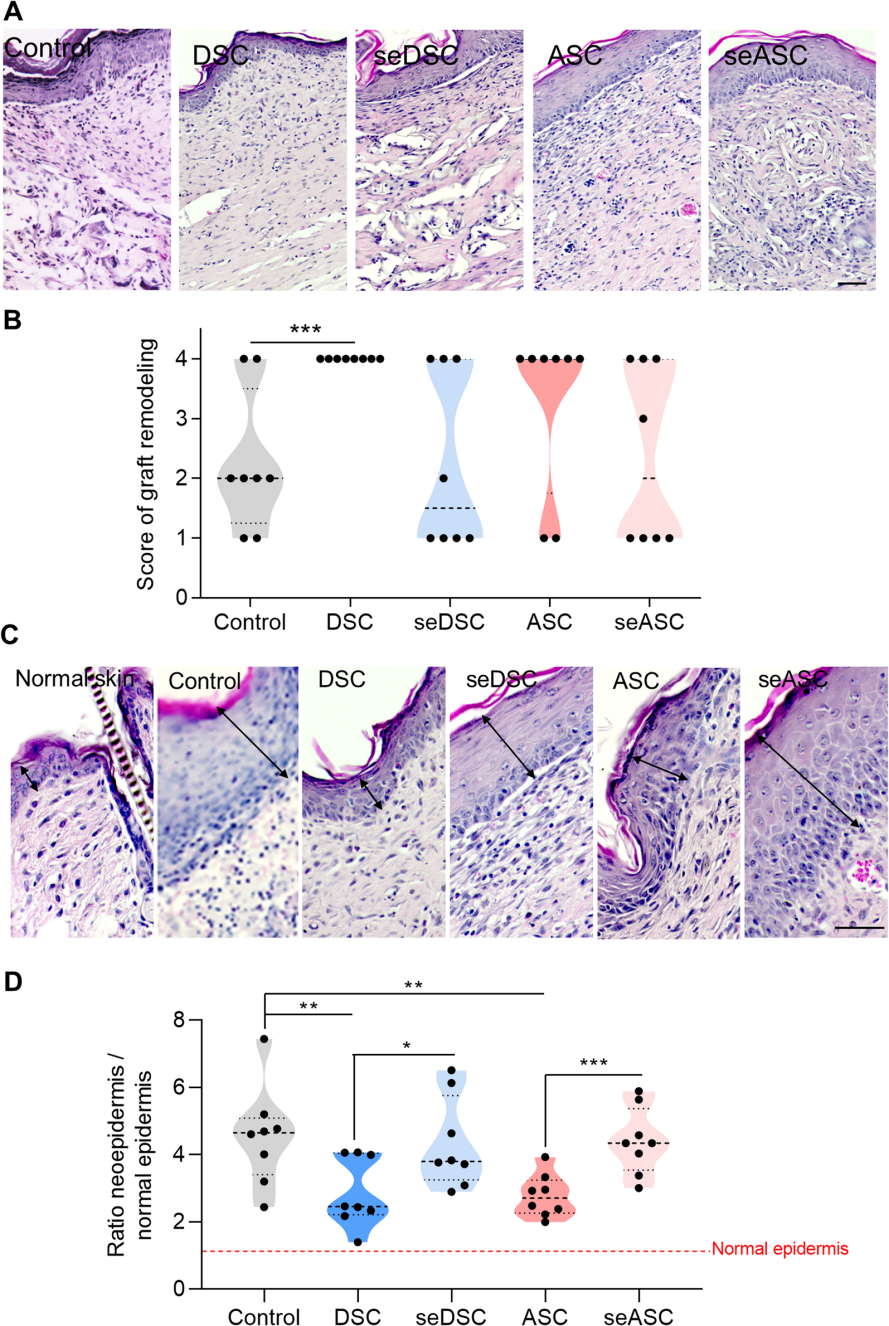
Matrix remodeling and reepithelization in cell- and secretome-treated wounds. **A** Representative images of graft collagen layer remodeling and **B** the corresponding quantification at day 21 post-wounding. Scale bar: 50 µm. **C** Representative images of the epidermis thickness in normal skin and scars. The black arrows indicate its thickness. Scale bar: 50 µm. **D** Ratio between neoepidermis and the normal epidermis thickness. The red dotted line shows the thickness value of the normal epidermis. Each data point represents the evaluation of one animal. *p < 0.05, **p < 0.01 and ***p < 0.001 by Student’s *t* test (control vs. treated groups; cells vs. secretomes groups)

The Integra Matrix collagen component supports the migration and proliferation of host cells during the inflammatory and proliferative phases of wound healing and is subsequently degraded by macrophages and replaced by the endogenous extracellular matrix during the remodeling stage of healing [13]. Thus, the degree of graft degradation/remodeling is an indicator of the maturity of the healing process. At day 21 post-wounding, DSC-treated animals had the graft completely degraded, with values statistically superior to the control group (*p* < 0.01), but not significantly different from those treated with ASCs or secretomes (Fig. 4A, B). Complete graft degradation was observed in 75% (six of eight) of ASC-, 37.5% (three of eight) of secretomes- and 25% (two of eight) of control-treated groups. These findings suggest that scaffold degradation was more pronounced in animals treated with cells than with their respective secretomes.

During remodeling, the initial hypertrophic and irregular neoepidermis formed in the wound site is reorganized and becomes more similar to the intact epidermis as the process matures [13, 47]. At day 21 post-wounding, the neoepidermis was thicker in empty scaffold control- and secretomes-treated animals than in animals treated with DSCs or ASCs (Fig. 4C, D). However, all groups had significantly greater neoepidermal thickness than the adjacent intact epidermis, although in different degrees. In cell-treated groups, the neoepidermis was 2.9- (DSC) and 2.8- (ASC) fold thicker than the intact epidermis, while in secretome-treated groups, it was 4.3- (seDSC) and 4.4- (seASC) fold that of the intact epidermis (control = 4.5-fold thicker). These findings indicate that secretomes are not as effective as cells in accelerating and promoting wound healing.

## Discussion

Large wounds cannot heal by primary or secondary intention and require grafts to close [48]. The first attempt to treat such wounds is made using autologous epidermal grafts harvested from the patient’s unaffected areas [49]. Aside from availability limitations, epidermal grafts alone invariably result in thin and weak scars, with poor elasticity and tensile strength due to the lack of a dermal component. Several materials engineered to serve as dermal templates have been developed and are FDA-approved for clinical use, such as the Integra Matrix [48, 50–52]. Although these strategies show a significant improvement over epidermal grafts alone, complications such as hematomas, seromas, infections, and graft detachment occur in about 50% of transplants, often requiring reapplications [53]. Besides, even when the procedure is successful, the result is a scar, i.e., a fibrotic tissue lacking complex skin structures and appendages, rather than fully functional regenerated skin [47]. Novel leading research approaches use engineered materials associated with stem or skin cells to promote wound closure and enhance scar quality. While outcomes are improved, clinical translation of strategies involving the transplantation of allogeneic and/or manipulated cells has been hampered due to safety concerns and extended FDA regulatory approvals required. Groundbreaking cell-free approaches based on cell-secreted bioactive factors avoid the hurdles of cellular therapies and are the most promising in regenerative medicine.

Our previous work established that the association of Integra Matrix with DSCs or ASCs promotes accelerated wound closure and improved healing in mice compared to empty scaffold controls [13]. We also established that DSC and ASC secretomes induce faster in vitro wound closure of human dermal fibroblasts and keratinocytes and increase vascular network formation in endothelial cells [7]. These studies suggested an influence of the source of MSCs in their therapeutic outcomes and highlighted the dermis as an overall superior source of MSCs for skin repair. Our findings led us to investigate further the particularities of DSC and ASC secretomes and their applicability for skin wound healing.

Herein, we characterized DSC and ASC secretomes regarding their protein components and tested their potential for skin wound healing. This work explored the dermis, an understudied source of MSCs and secreted factors for skin repair, and is the first study to show that secretomes obtained using standard methods are not as effective as the cells they derive from in promoting skin wound healing.

First, evaluation of seDSC and seASC protein content revealed a plethora of factors involved in tissue repair. We found several important proteins involved in angiogenesis, such as TGFB1, ANGPT1, MMP2/9, and TIMP1, which may explain the promotion of tubule formation in vitro we previously described [7], and the increase in blood vessel density in seDSC and seASC-treated wounds we reported herein. These findings corroborate with previous studies by our group and others, where DSC and ASC secretomes were independently investigated [54, 55].

Interestingly, although seDSC and seASC showed different expression of several proteins, many of them display similar or redundant roles in wound healing. For example, both secretomes were enriched in members of IGFBP family, proteins that modulate IGF1; however, while IGFBP2 was present only in seDSC, IGFBP5 was exclusive of seASC. Several serpins were also present in the secretomes, but Serpin A1 was increased in seDSC and Serpin A2 was increased in seASC. Additionally, while only seDSC expressed IQGAP2 and PCDH7, the proteins LAMA1 and ECM2 were only found in seASC, and all these factors have modulatory roles in cell adhesion. Particularities observed in each secretome composition support an influence of the tissue source on overall MSC protein expression and could play a role in DSC and ASC's distinct potentials for skin wound healing [7, 13]. However, we surprisingly did not detect any significant differences between mouse wounds treated with seDSC or seASC. Our findings suggest that DSC and ASC secretomes may promote similar effects on wound healing but through distinct pathways. The high complexity of MSC secretomes composition explain why the mechanisms behind MSC and secretome therapeutic effects remain poorly elucidated.

The rapid closure of skin wounds plays a crucial role in preventing the entry of microorganisms and minimizing fluid loss from the body [56]. Contrary to the notable acceleration in wound closure observed by DSC and ASC treatments, the secretomes did not promote a significant effect compared to the empty scaffold. Accordingly, the granulation tissue formed in the proliferative phase of healing was less developed in secretome-treated groups than in cell-treated groups, suggesting an impaired ability to induce fibroblast proliferation and extracellular matrix production. At the remodeling phase, secretome-treated wounds showed delayed maturation of the neoepidermis and graft degradation compared to cell-treated groups. Nevertheless, secretomes successfully stimulated graft integration and angiogenesis, two critical processes to ensure adequate graft implantation. Overall, our data show that MSC secretome treatments lead to intermediary results in comparison to cells and empty scaffold controls.

Although the beneficial effect of MSC secretomes on wound closure compared to untreated controls has been previously demonstrated, comparison with the cells they originate from is severely lacking [54, 57, 58]. The few studies that made such a comparison (in lung injury and ovarian failure models) corroborate our findings [59, 60]. As transplanted cells secrete paracrine factors in response to their microenvironment [61–63], it is understandable that the secretome collected from cells cultured under artificial, standard conditions could not promote equally effective outcomes. Therefore, the superior healing properties of MSCs observed in our study may be explained by their secretion of paracrine factors in response to cues from the wound environment, while in culture, MSCs lack such stimulus. Additionally, the limited secretome effectivity could also result from an insufficient dose, as this study did not intend to compare different dosages and single versus repeated applications. The dose used here was previously proven effective in our in vitro studies of wound healing (cell scratch) and vascular network formation [7]. Nevertheless, there are currently no scientific consensus regarding secretome doses and number of applications and it is possible that further optimization may be needed for successfully treating mouse wounds.

Recently, MSC preconditioning started to be tested as a way to improve secretome quality. It has been shown that MSC incubation in hypoxia leads to increased secretion of angiogenic factors (FGF2, VEGF, TGFβ, angiogenin, TIMP1, CCL20, MCP1, MMP9, miR-210, miR-125b-5p, miR-126, miR-130a, and miR-210) and greater angiogenesis in vitro and in vivo [64–66]. Likewise, MSCs cultured with the inflammatory cytokines TNFα, IFNγ, or IL1α produced secretomes with improved anti-inflammatory effects as assessed by inhibition of T-cell proliferation [65–67]. However, preconditioned secretomes have not been compared against the cells they derive from. Therefore, it is still unclear if such approaches would effectively substitute cellular therapies. Additionally, given the complexity and multifactorial nature of wound healing, together with the diversity of wound etiologies and patient comorbidities, individual factors are unlikely to induce a comprehensive response.

## Conclusion

In summary, this study revealed that seDSC and seASC promote intermediary effects in comparison to cells and empty scaffold controls, as evaluated by wound closure and histological analysis of angiogenesis and granulation tissue formation at the proliferative phase, and scar maturation at the remodeling phase of wound healing. Our data indicate that the secretomes obtained from standard culture conditions are not able to reproduce MSC effects on wound healing and highlight the need for further investigation in MSC preconditioning and secretome modulation for improved outcomes.

### Supplementary Information

**Additional file 1**. SeDSC and seASC proteomics and gene ontology.

## Data Availability

All data generated during this study are included in this published article and its supplementary information files. The mass spectrometry proteomics data have been deposited to the ProteomeXchange Consortium via the PRIDE partner repository with the dataset identifier PXD047650.

## Abbreviations

ABI3BP: ABI family member 3-binding protein
ANGPT1: Angiopoietin-1
ASCs: MSCs derived from the human adipose tissue
A2M: Alpha-2-macroglobulin
BMP: Bone morphogenetic protein
CCBE1: Collagen and calcium-binding EGF domain-containing protein 1
CFL1: Cofilin-1
CHI3L1: Chitinase-3-like protein 1
COL15A1: Collagen alpha-1 (XV) chain—restin 1,2,3,4
COL5A1: Collagen alpha-1 (V) chain
COMP: Cartilage oligomeric matrix protein
DDX42: ATP-dependent RNA helicase
DMEM: Dulbecco's Modified Eagle's Medium
DSCs: MSCs derived from the human dermis
ECM2: Extracellular matrix protein 2
EIF3D: Eukaryotic translation initiation factor 3 subunit D
FERMT2: Fermitin family homolog 2
GO: Gene ontology
GREM1/2: Gremlin 1/2
HSPG2: Heparin sulfate proteoglycan core protein
IGFBP: Insulin-like growth factor-binding protein
IL: Interleukin
IQGAP2: Ras GTPase-activating-like protein
LAMA1: Laminin alpha 1 subunit
LC–MS/MS: Liquid chromatography with tandem mass spectrometry
LFQ: Label-free quantification
MMP: Matrix metalloproteinase
MSCs: Mesenchymal stem/stromal cells
NOV: Nephroblastoma overexpressed protein
PARP1: Poly [ADP-ribose] polymerase 1
PCDH7: Protocadherin-7
PLAU: Urokinase-type plasminogen activator
PTGIS: Prostacyclin synthase
RARRES2: Retinoic acid receptor responder 2 protein
SBSN: Suprabasin
seASC: Adipose-derived MSC secretome
seDSC: Dermal-derived MSC secretome
SEMA7A: Semaphorin-7A
SERPINE1: Serpin family E member 1
SERPINE2: Serpin family E member 2
SFB: Fetal bovine serum
SLIT2: Slit homolog 2 protein
SPARC: Secreted protein acidic and rich in cysteine
TGFB1: Transforming growth factor beta 1
THBS2: Thrombospondin 2
TIMP1: Tissue inhibitor of metalloproteinases 1
VCAN: Versican core protein
VCL: Vinculin
