# Correction: Evaluation of secretomes derived from human dermal and adipose tissue mesenchymal stem/stromal cells for skin wound healing: not as effective as cells

**DOI:** 10.1186/s13287-024-03697-1

**Published:** 2024-03-18

**Authors:** Helena Debiazi Zomer, Victor Juan de Souza Lima, Monique Coelho Bion, Karynne Nazare Lins Brito, Michele Rode, Marco Augusto Stimamiglio, Talita da Silva Jeremias, Andrea Gonçalves Trentin

**Affiliations:** 1 Department of Physiological Sciences, University of Florida, Gainesville, USA; 2 Department of Cell Biology, Embryology, and Genetics, Federal University of Santa Catarina, Florianópolis, Brazil; 3 National Institute of Translational Neuroscience, Federal University of Rio de Janeiro, Rio de Janeiro, Brazil; 4 Laboratory for Stem Cells Basic Biology, Carlos Chagas Institute, FIOCRUZ/PR, Curitiba, Paraná Brazil; 5 National Institute of Science and Technology for Regenerative Medicine, Rio de Janeiro, Brazil

**Correction: Stem Cell Research & Therapy (2024) 15:15**

10.1186/s13287-023-03630-y

The original article initially contained an error in Fig. 1A whereby the Venn diagram overlapped the text. This has since been corrected.

